# Boosting the thermal management performance of a PCM-based module using novel metallic pin fin geometries: Numerical study

**DOI:** 10.1038/s41598-023-37639-3

**Published:** 2023-07-06

**Authors:** Abdelrahman M. Elshaer, A. M. A. Soliman, M. Kassab, A. A. Hawwash

**Affiliations:** 1 Mechanical Engineering Department, Benha Faculty of Engineering, Benha University, Benha, Egypt; 2 Egyptian Space Agency, New Cairo, Egypt; 3 Faculty of Engineering and Technology, Badr University in Cairo, Cairo, Egypt

**Keywords:** Engineering, Mechanical engineering

## Abstract

Satellite avionics and electronic components are getting compact and have high power density. Thermal management systems are essential for their optimal operational performance and survival. Thermal management systems keep the electronic components within a safe temperature range. Phase change materials (PCMs) have high thermal capacity, so they are promising for thermal control applications. This work adopted a PCM-integrated thermal control device (TCD) to manage the small satellite subsystems under zero gravity conditions thermally. The TCD's outer dimensions were selected upon a typical small satellite subsystem. The PCM adopted was the organic PCM of RT 35. Pin fins with different geometries were adopted to boost the lower thermal conductivity of the PCM. Six-pin fins geometries were used. First, the conventional geometries were square, circular, and triangular. Second, the novel geometries were cross-shaped, I-shaped, and V-shaped fins. The fins were designed at two-volume fractions of 20% and 50%. The electronic subsystem was assumed to be "ON" for 10 min releasing 20 W of heat, and "OFF" for 80 min. The findings show a remarkable decrease in the TCD's base plate temperature by 5.7 ℃ as the fins' number changed from 15 to 80 for square fins. The results also show that the novel cross-shaped, I-shaped, and V-shaped pin fins could significantly enhance thermal performance. The cross-shaped, I-shaped, and V-shaped reported a decrease in the temperature by about 1.6%, 2.6%, and 6.6%, respectively, relative to the circular fin geometry. V-shaped fins could also increase the PCM melt fraction by 32.3%.

## Introduction

**Figure 1:**
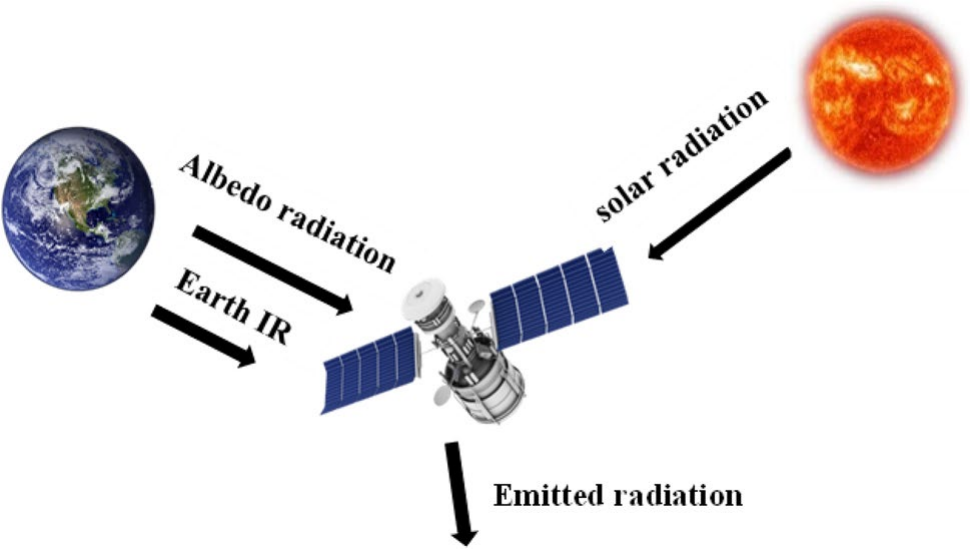
Heat sources the spacecraft encounters in space.

Low earth orbit (LEO) satellites are exposed to a harsh thermal environment. Satellites in LEO are sometimes in the hot zone and other times in the cold zone. High heat is obtained from the sun during the hot zone, resulting in overheating satellite components. But, in the cold zone, there is a lack of heat energy which causes significant temperature drops in satellite subsystems. Figure 1 presents the sources of heat that the spacecraft encounters in orbit: solar radiation, earth's diffusive radiation (Albedo), planet's emission of infrared radiation, friction between gas molecules and spacecraft body in the space environment, and avionics systems' energy dissipated^1^.

The satellite thermal control subsystem manages the temperature of the other subsystems. The satellite subsystems have a recommended temperature range within which their temperature values lie. Any additional heating or cooling of any subsystem can significantly reduce the performance and lead to complete failure. Electronic devices and associated components used in the spacecraft industry, particularly satellite subsystems, have recently become more compact and have higher power density. These electronic gadgets and satellite subsystems also use more electricity. Consequently, novel technologies are urgently required for effective thermal control of satellite components^2^.

The satellite subsystems operate cyclically; they are "ON", sometimes releasing thermal energy and "OFF" with shut down mode. Small satellites' thermal management is complex due to mass limitations, small heat capacity, and power source limitations. Satellite thermal control systems are classified into two main categories: passive and active thermal control subsystems. On the one hand, the passive thermal control subsystems require no electrical power, such as radiators, insulation, etc. On the other hand, the active thermal control subsystems require electrical power. Active thermal control subsystems include fluid-looped systems, heaters, coolers, etc.

Thermal energy storage materials (TES) materials are a good choice for small satellites' thermal management in the LEO thermal environment. Three types of TES materials exist sensible energy storage (SES), latent energy storage (LES) and chemical energy storage materials. The former materials have higher specific heat values, while the latter stores the heat by changing its physical phase, called phase change materials (PCMs). The most common PCM are the solid–liquid PCMs. Solid–liquid PCMs store heat energy during heating, and their phase changes from solid to liquid. The stored heat energy is rereleased during cooling, and the phase returns to the solidus state^1^.

## Literature review

Numerous studies presented the viability of utilising PCM in electronics thermal control. Humphries^3^ examined how PCM change their phase in normal and zero-gravity environments. Abhat et al.^4^ investigated using PCM filler materials of honeycomb shape in a PCM-based thermal control device (TCD). The results demonstrated the ability of a honeycomb architecture to keep hardware temperatures within restricted bands, which was essential for satellite operation. Furthermore, the authors reported that natural convection impacts in the PCM were negligible for space applications.

Desai et al.^5^ studied the effect of inverting fins on enhancing the thermal management performance of a PCM-integrated thermal management device. A thermal control unit (TCU) based on PCMs was examined to manage the maximum temperature of an electronic component. The results reported a decrease in peak temperature achieved with the inverted fins by 9%. A numerical study was delivered by Kansara et al.^6^ investigating the effect of the heat source direction on the thermal management performance of a heat sink integrated with PCM. The melting process of the PCM was studied at different values of gravitational acceleration varying from g to (1/80) g. The results concluded that the inverting of the direction of the heat source remarkably affects the performance of the PCM-integrated TCU.

Pakrouh et al.^7^ reported a numerical study on fin shape optimisation integrated with PCM-based heat sinks. Organic PCM of Paraffin RT44 hydrocarbon (HC) was used. At the same time, the heat sink base and fins were designed of aluminium. The findings showed a complex interrelation between the PCM and the PCM thermal conductivity booster (TCB) volume fractions. An experimental study was performed by Fan et al.^8^. The study examined the effects of the PCM melting point and fins on the thermal performance of a PCM-integrated heat sink for the thermal management of electronic components. The findings presented that the PCM of lower melting temperature was possibly favoured. In addition, using internal fins was preferred as the heat sink's performance improved.

Fan et al.^9^ experimentally studied the impact of using carbon nano-fillers with high aspect ratios to improve the thermal conductivity of the PCM. The results reported a remarkable improvement in the thermal performance by using PCM/ carbon nanofillers composite. Gharbi et al.^10^ compared different geometries of PCM-integrated heat sinks for cooling electronic devices. The results revealed that combining the PCM with fins greatly enhanced thermal management performance. Levin et al.^11^ conducted a numerical study for the thermal optimisation of a PCM- integrated heat sink. The study showed that optimal PCM percentages depend on the fins length, the fins count, the heat flux, and the integrated fins interface with PCM.

Al-Abidi et al.^12^ incorporated internal and external fins for heat transfer enhancement of a TES system using PCM. The results showed an enhancement in the PCM melting time by 34.7% when a PCM unit with 8-cell geometry was adopted. Hosseini et al.^13^ delivered an experimental and numerical study to investigate the melting behaviour of PCM inside shell and tube heat exchangers. The working fluid's inlet temperature effect was examined against the PCM's melting process. Tharwan et al.^14^ investigated experimentally the thermal performance of a PCM-integrated heat sink. The findings reported a remarkable enhancement in the heat sink thermal performance using PCM. In addition, Akula et al.^15^ conducted both experimental and numerical investigations to study the thermal performance of PCM/fins/expanded graphite (EG) composites for thermally managing lithium-ion batteries. The results revealed a significant enhancement in the thermal performance by integrating the EG and fins within the PCM. The additives could improve the PCM thermal conductivity significantly. Manoj Kumar et al.^16^ performed an experimental analysis studying the influence of nano-improved PCMs on cooling electronics. Nano-silica/ PCM composite was adopted. The study concluded that nano-improved PCM could improve thermal performance significantly.

Jalil et al.^17^ presented an experimental and numerical study investigating the impact of PCM on the cooling performance of a heat sink. Then the effect of adding nanoparticles to PCM was also investigated. It was reported that a low percentage of nanoparticles (2%) drastically improved the heat sink's performance. A practical study reported by Ding et al.^18^ studied the contribution percentage of natural convection effects in a PCM-integrated heat sink by altering the location of the heat source. The findings concluded that the master heat transfer mode was natural convection rather than thermal conduction for the whole melting process of PCM under fluctuating heat load. Huang et al.^19^ delivered an experimental and numerical study evaluating a new cascaded low melting point alloy PCM-integrated heat sink. The study showed that the PCM volume fractions and configuration significantly influence the thermal performance of the cascaded PCM-based heat sinks.

Arshad et al.^20^ delivered a parametric study investigating a PCM-based heat sink with flat plate fins for the thermal management of electronics. The study revealed that a PCM/ flat plate fins composite remarkably improved the PCM melting and significantly decreased the base plate temperature. Al-Omari et al.^21^ delivered a numerical study proposing novel fins concept with lifted ends from the heat sink bottom hot base. Findings s reported that the fin-lifting idea could lead to a significant decrease in temperatures. In addition, the novel fin system resulted in a considerable increase in the heat loss rate from the heat sink. A numerical model was conducted by Huang et al.^22^ to investigate a novel dual- PCM-integrated heat sink by adopting both paraffin and low melting point alloys. Results concluded that increasing the volume fraction of low melting point PCM can significantly extend the melting time and the second melting stage, improving the synchronisation of the melting process. A numerical model designed by Ghanbarpour et al.^23^ analysed the thermal performance of a proposed heat sink using PCM and vapour for cooling applications. The model findings concluded that the fin height and count were more influential than the fin thickness in decreasing the temperature of the heat source.

Wang et al.^24^ experimentally explored heat sinks' thermal performance by adopting higher alcohol alone and higher alcohol/graphite foam composite as a PCM. The results showed that a higher alcohol/graphite foam composite-integrated heat sink improved the thermal performance of a higher alcohol-integrated heat sink. Hu et al.^25^ investigated a porous material used as TCB, designed and manufactured using the three-dimensional printing methods. The study revealed a significant improvement in the thermal response of heat sinks for cooling electronic systems. Bondareva et al.^26^ conducted a numerical study investigating the impact of the copper profile geometric configuration on lauric acid phase change with a volumetric heat generation device. The findings reported that fins of vertical plate shape boost thermal diffusion faster than those of horizontal shape within the metallic structure. Kalbasi^27^ reported a novel heat sink filled with hybrid PCM/air composite for managing the electronic components thermally. The finding showed that the cooling ability of the novel PCM/air-cooled heat sinks was better than the PCM-based heat sinks. Al-Omari et al.^28^ conducted a numerical analysis of a proposed new fins concept with lifted ends from the base of a heat sink. Results showed that the fins lifting technique could reduce the peak temperatures significantly and increase the heat discharge rate from the heat sink.

Fauzi et al.^29^ delivered an experimental study investigating the effects of adding an acidic surfactant in improving the thermophysical properties of eutectic PCM. It was concluded that adding 10% sodium laurate to the eutectic PCM significantly decreased the phase change temperature. A numerical study by Nedumaran et al.^30^ investigated the impacts of fins orientation as a TCB on the performance of a PCM-integrated heat sink. The study showed that the multiple PCM arrangements in the cavity do not significantly influence the melting time. Nayak et al.^31^ studied the impact of TCBs numerically in boosting the overall thermal conductance of PCM for electronics cooling. The results illustrated a significant and favourable effect of the TCB on thermal performance. Hubert et al.^32^ delivered an experimental and numerical study investigating the effective thermal conductivity of porous architected structures integrated with PCM. The study reported that the architected structure had an effective thermal conductivity 75% higher than the TCB of foam at the same porosity. Choi et al.^33^ investigated experimentally the thermal conductivity of PCM/Multi-walled Carbon nanotubes (MWCNTs), carbon additives, graphite, and graphene composite. Graphite was reported to be an up-and-coming candidate for enhancing the heat transfer process.

Alshaer et al.^34^ studied the impact of integrating carbon foam and MWCNTs on the thermal management performance of a PCM-based TCD. The results showed a remarkable enhancement in the PCM/carbon foam thermal control performance and PCM/MWCNTs composite. Arshad et al.^35^ studied experimentally the PCM-integrated heat sinks with pin–fin for the passive thermal management of electronic devices. The optimal thermal performance in operating time was obtained for the pin–fin with 2 mm thick, as reported in conclusion. Fu et al.^36^ examined the PCM experimentally and expanded graphite as a thermal conductivity enhancer for floor heating systems. The results indicated that the composite PCM was an up-and-promising candidate for heat exchangers. Nada et al.^37^ investigated the PCM/MWCNT composite experimentally for energy storage systems. The results concluded that MWCNT increased the PCM thermal conductivity significantly.

Raj et al.^38^ investigated the effect of fin configuration on heat transfer for satellite thermal management. The study reported the higher efficiency of tapered fin geometries over straight fins. Kansara et al.^39^ addressed the effect of gravity on the melting and solidification process of PCM. The study concluded that the PCM liquid fraction decreased by 18% under low gravity conditions. Elshaer et al.^40^ studied numerically a PCM-based heat sink for thermal control of a small satellite subsystem using metal foam. The findings showed that the novel metal foam geometry drastically enhanced thermal performance and brought the lowest temperature. Parsa et al.^41^ revealed the most efficient side of thermoelectric in two solar stills comparatively using turbulator, PCM, and nano-paint. The findings showed that thermoelectric heating systems had about 20% and 7% daily energy exergy efficiency higher than thermoelectric cooling-system. Saeed et al.^42^ investigated electronics thermal management by forced air cooling technique of fire avoidance. The study concluded that the cross-flow configuration performs better than the reverse flow. Ali^43^ developed a review discussing the recent research in electric vehicles' battery thermal management. In addition, Kumar Thakur et al.^44^ presented a review article summarising the state of the art in the battery thermal management of fast charging systems. Elshaer et al.^45^ studied the effect of PCM combination on the thermal management performance of satellites in LEO intermittent environment. The findings revealed that the thermal performance for PCM combinations was highly independent of the heating load relative to the single PCM cases.

## Critical summary and the aim of the present work

As surveyed in the former literature, more research showed interest in combining PCM and metallic fins for heat transfer enhancement. Many researchers have proven the efficacy of PCM/fins combination in thermal management applications. Different applications were studied, such as thermal control of electric vehicles, lithium-ion batteries, electronics, and renewable energy systems. Part of the studies investigated the influence of fin count, and others examined the impact of fin geometry on thermal performance. Based on the authors' best survey, no previous study was performed numerically to investigate the novel pin fins geometries of cross-shaped, I-shaped, and V-shaped geometries on a PCM-integrated TCD. The current research presents novel pin fin geometries as TCBs integrated into a PCM-integrated heat sink as a TCD. The TCD was required to manage a small satellite subsystem thermally. The outer size of the device was based on a typical small satellite subsystem of 100 × 70 × 20 mm^3^. The satellite subsystem worked intermittently in a thermal cycle for 10 min releasing 20 W thermal heat, and for 80 min, switched off, releasing no heat energy.

Six-pin fin geometries were adopted in the present work. The conventional geometries of the square, circular, and triangular geometries were adopted. Second, the novel pin fin geometries of cross-shaped, I-shaped, and V-shaped geometries were adopted. RT 35 was used as the PCM in this study. The fins were modelled based on two TCBs volume fractions of 20% and 50%. The TCD was supposed to work in space. Thus, the convection heat transfer from the heat sink outside surfaces was neglected, and only the radiation heat transfer was considered in this study. A three-dimensional pin fins/PCM composite model was built and simulated using ANSYS fluent. The present work investigated the effects of varying the fins count and the fin geometry effects. The number of fins ranged from 15 fins up to 80 fins. The TCD's thermal performance was analysed using three factors: the temperature of the base plate, PCM melt fraction, and the critical time for which the device temperatures exceeded the critical ones. The novelty and importance of this work are outlined as the following.

- A TCD with an outer size based on a typical small satellite subsystem was designed and tested for the thermal management of small satellites.
- The study discusses one of the methods for enhancing PCM thermal conductivity: metallic fins integration with PCM. The study provides novel shapes of fins to provide innovative solutions for further boosting the thermal management performance of TCD.
- Novel pin fin geometries of cross-shaped, I-shaped, and V-shaped geometries were combined with a PCM-integrated TCD.
- The TCD was analysed using a three-dimensional model under microgravity conditions.
- The TCD was required to manage a small satellite subsystem thermally and tested under intermittent thermal conditions.

## Model

The model and the associated materials are presented in this section. “The model configurations and materials” presents the model's design, model configurations, and materials. In addition, “Physical problem statement and the boundary conditions” discusses the physical problem and initial and boundary conditions. “The governing equations” presents the governing equations.

### The model configurations and materials

An aluminium heat sink was used as a TCD to manage a small satellite subsystem thermally. The outer size of the TCD was based on a typical satellite subsystem with external dimensions of 100 × 70 × 20 mm^3^. The TCD walls and base plate thickness was 5 mm for all. RT 35 was used as a PCM^46^. The used PCM was organic-based because it shows higher thermal stability when exposed to repeated phase-change processes. The thermophysical properties of the materials used in the current work are listed in Table 1**.**

**Table 1:** Materials properties^43^.

Materials	Melting temperature (℃)	ρ (kg/${\mathrm{m}}^{3})$		C_p_ (J/kg)	k (W/m. K)	L (J/kg)
		Solid	Liquid			
RT 35	34–35	862	771	2050	0.21	158,000
Aluminium alloy (6061 T6)	–	2760	–	895	167.5	–

The PCM height in the TCD was 15 mm in all cases. As a result, the volume expansion of the PCM was disregarded. To overcome the low PCM thermal conductivity, aluminium pin fins were included in the TCD cavity. Six-pin fins geometries were investigated: triangular, square, circular, cross-shaped, I-shaped, and V-shaped. The pin fin geometries were built at 20% and 50% TCB volume fractions. TCB volume percentage is the fins volume to the PCM volume, as shown in Eq. (1). The contact area enhancement was calculated from Eq. (4).

1 $$\Psi =\frac{{V}_{fins}}{{V}_{PCM}}$$

2 $${V}_{fins}=\frac{\Psi {.V}_{t}}{1+\Psi }$$

3 $${V}_{PCM}=\frac{{V}_{t}}{1+\Psi }$$

where $\Psi$ is the fins volume fraction, and ${V}_{t}$ is the total volume of PCM and fins.

4 $$\mathrm{Contact \, area \, enhancement \, ratio}=\frac{N{A}_{fin}+{A}_{walls}}{{A}_{walls}}$$

Where N is the number of fins, ${A}_{fin}$ is the fin side surface area, ${A}_{walls}$ is the surface area of internal walls (side walls and bottom plate).

**Figure 2:**
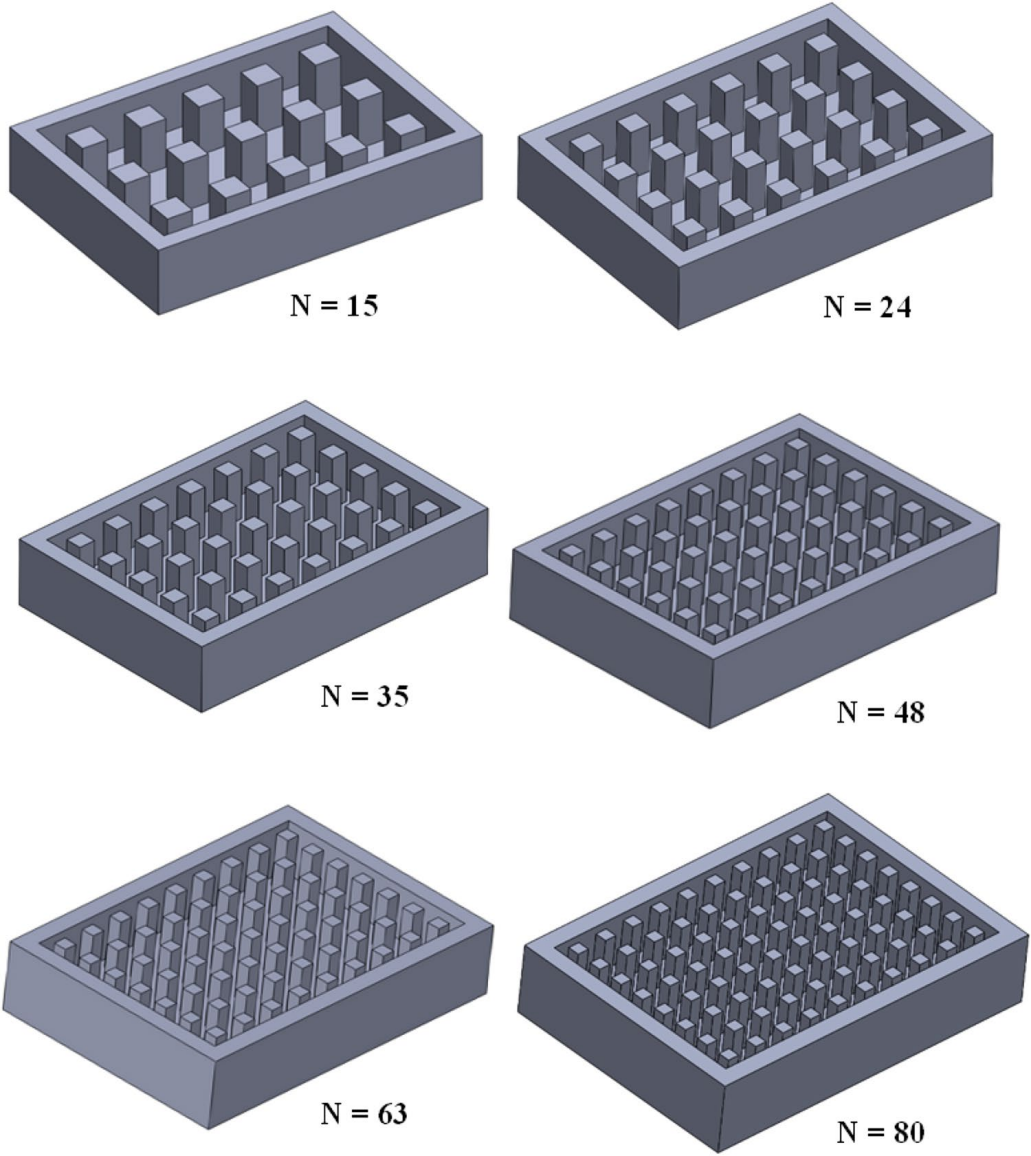
TCD with square pin fin geometry at a different number of fins and $\Psi$ = 20%.

**Figure 3:**
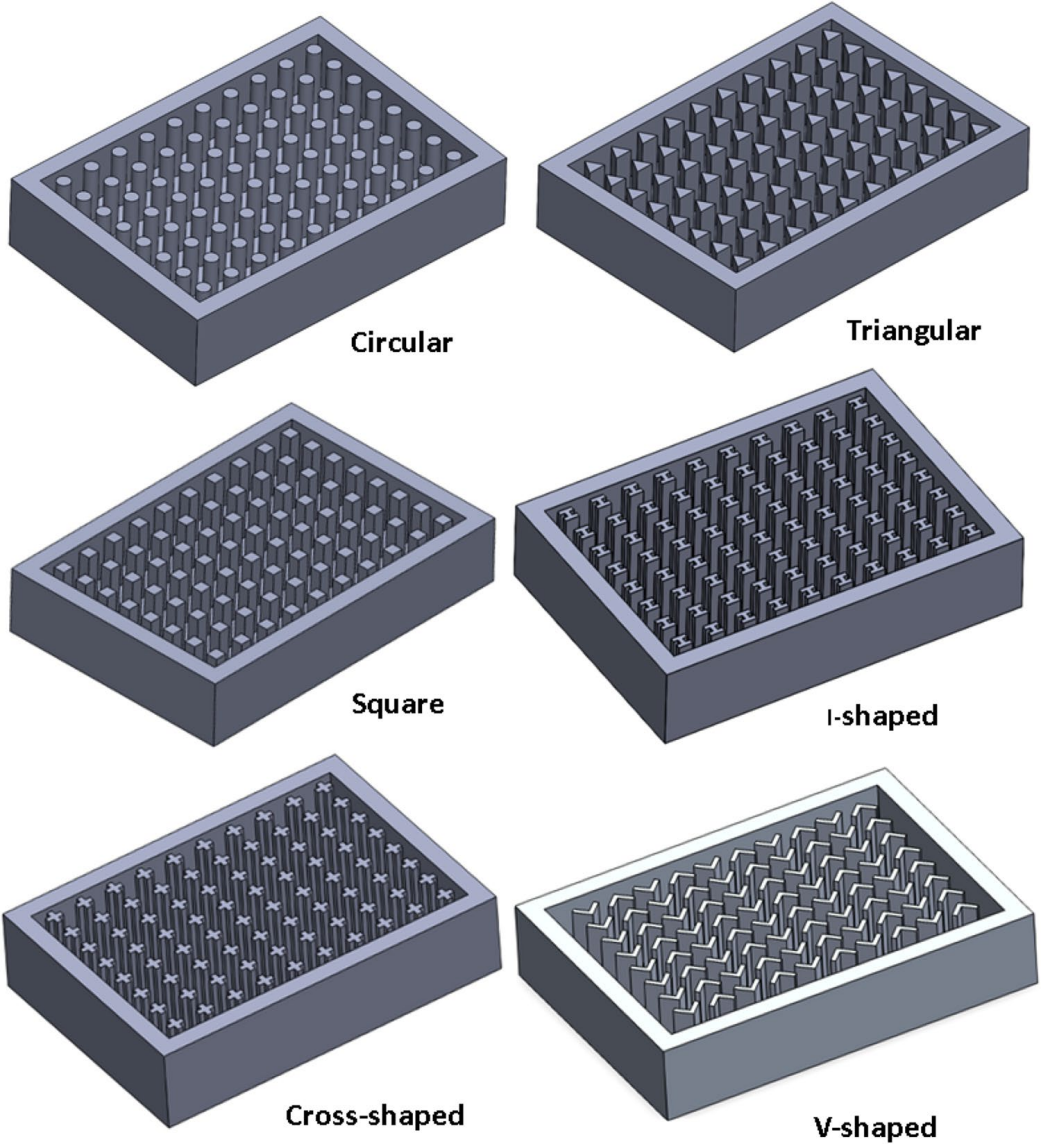
Pin fins geometries investigated in the present work at $\Psi$ = 20% and N = 80 fins.

**Figure 4:**
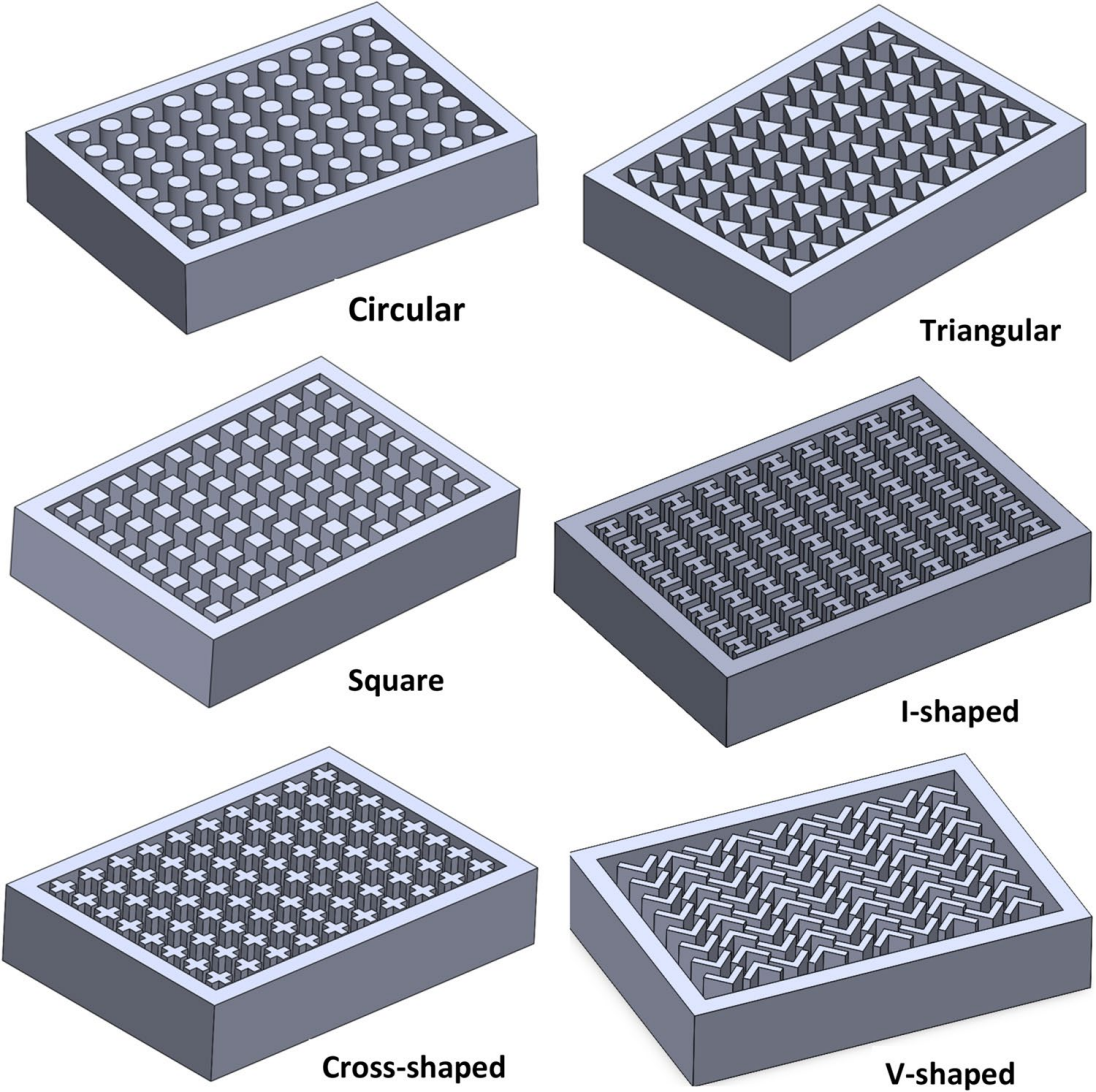
Pin fins geometries investigated in the present work at $\Psi$ = 50% and N = 80 fins.

Pin fins volume was 13,500 mm^3^ and 27,000 mm^3^ at 20% and 50% fractions, respectively. The TCD's cover plate was coated with a highly emissive black coating with a thermal emissivity 0.82. The cover plate was considered a thermal radiator that rejects the incident heat to the surrounding space by radiation. The current study investigated the TCD thermal control performance at different fins geometry and number. The effect of fins number was carried out using square fin geometry at a 20% TCB volume fraction. Figure 2 shows the geometries of square pin fins, with the count varying from 15 to 80 fins. While Figs. 3 and 4 present the fin geometries adopted in the current research at TCB volume fractions of 20% and 50%, respectively. For more details about the dimensions and configurations of the TCD with all fins geometries, see Appendix (A).

### Physical problem statement and the boundary conditions

**Figure 5:**
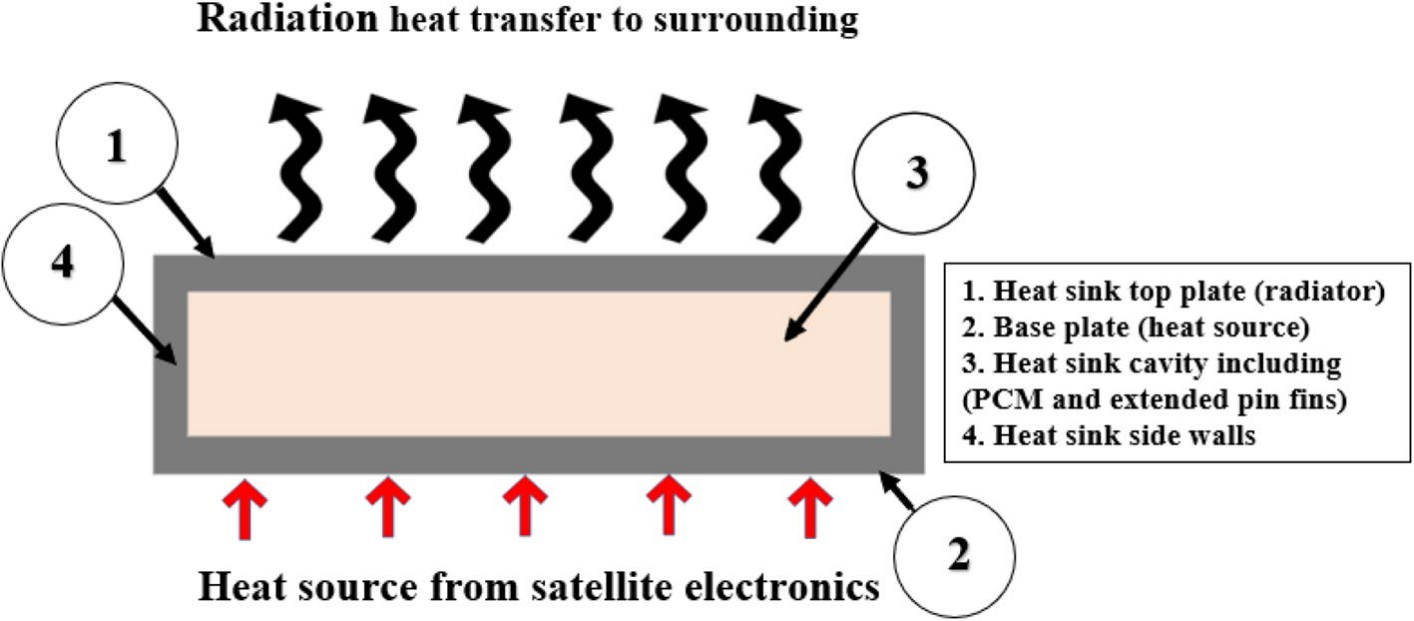
Physical problem description and model elements.

**Figure 6:**
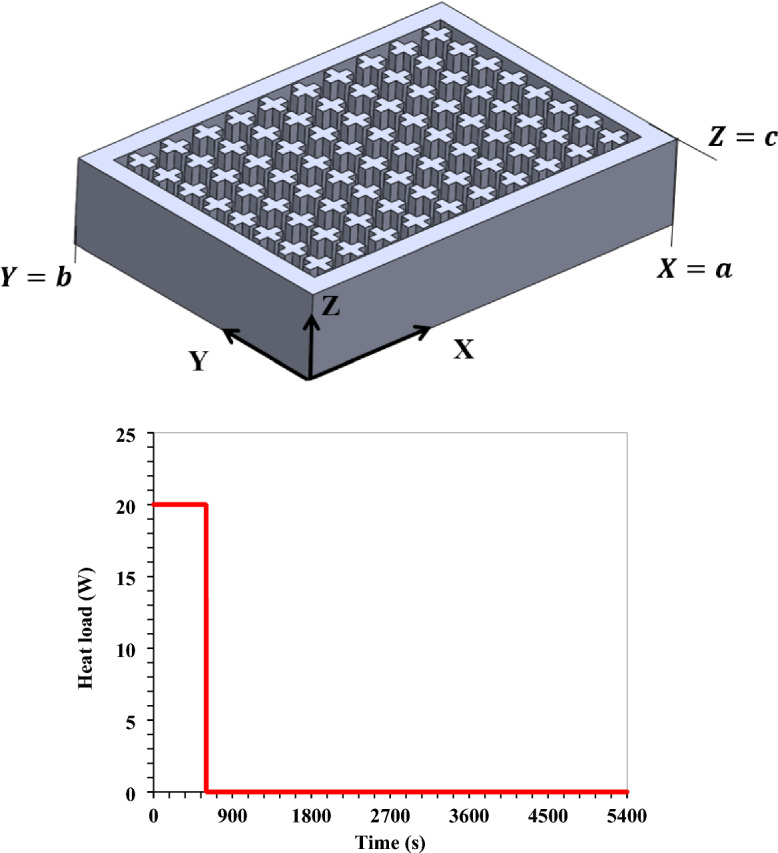
Heat load cycle of the satellite hardware and the adopted reference coordinate system for boundary conditions.

A 3D model of the PCM-integrated TCD was investigated using the ANSYS Fluent 2020 R1 solver. The avionics heated the TCD at the base plate. The duration of the thermal analysis cycle was 90 min in total. The satellite subsystem was in (ON) mode during the heating phase for 10 min. In contrast, it was in the (OFF) state throughout the 80 min cooling operation. The heat load on the satellite's hardware was 20 W (2857.1 W/m^2^). During the cooling phase, the TCD emitted thermal radiation to release the thermal energy it had received. The heat was radiated from the TCD's surface to a surrounding temperature of 20 °C. The model components and the physical problem statement are depicted in Fig. 5. At the same time, Fig. 6 displays the heat load of the satellite hardware during the cycle.

Some assumptions were considered in the numerical model for minimising the mathematical model's complexity.

1. During the simulation, the PCMs' properties for solid and liquid phases were constant and temperature independent.
2. PCMs were isotropic, homogenous, and incompressible materials.
3. The PCM phase change volume expansion was disregarded due to the negligible effect on the thermal performance results, as stated in previous literature^26,38,47^.
4. The difference between liquidus and solidus temperatures was 1 °C^47,48^.
5. The primary mode of heat transfer was conduction; furthermore, the natural convection effects within the PCM were disregarded^49–51^.
6. Convection heat transfer mode and gravity effects were disregarded as working in space.

The TCD underwent heating and cooling processes in the solution cycle. It was heated for 10 min using the satellite hardware component's heating load at the bottom plate. The hardware component was switched off, and the TCD emitted heat radiation to the surrounding at 25 °C during the cooling process. The cooling procedure lasted for 80 min. The initial cycle temperature was set to 22 °C, and the sidewalls were insulated. The following are the details of the initial and boundary conditions, respectively.

1. The Initial conditions at (t = 0 s)
  $${T}_{i}$$= 22 ℃.
2. Boundary conditions.
  - Heat flux at the bottom plate
    At $$(0\le t<600) \mathrm{s}$$
    $${q}_{x,y,0,t}={q}_{hardware}=20 \mathrm{W}$$
    At $$(600\le t\le 5400) \mathrm{s}$$
    5 $${q}_{x,y,0,t}=0$$
  - (b) Insulated side walls
    6 $${\left.\frac{\partial T}{\partial x}\right|}_{(0,y,z,t)}=0$$
    7 $${\left.\frac{\partial T}{\partial x}\right|}_{(a,y,z,t)}=0$$
    8 $${\left.\frac{\partial T}{\partial x}\right|}_{(x,0,z,t)}=0$$
    9 $${\left.\frac{\partial T}{\partial x}\right|}_{(x,b,z,t)}=0$$
  - (c) Emitted radiation from the top plate
    At $$(0\le t\le 5400) \mathrm{s}$$
    10 $${q}_{(x,y,H,t)}=-{q}_{rad.}$$
    where ${q}_{rad.}$: is the radiation heat transfer as Eq. (11). The surrounding temperature was assumed constant during the entire orbit of 25 ℃^2^.
    11 $${q}_{rad}=\varepsilon \sigma {A}_{s}{({T}_{surface}}^{4}-{T}_{\infty }^{4})$$

### The governing equations

The finite volume method was adopted to conduct the numerical simulations on the TCD model. Modelling the phase change process was achieved using the enthalpy-porosity approach. In the enthalpy-porosity approach, each cell was considered a porous domain. This domain's porosity corresponds to the PCM's melt fraction in the domain^52,53^. The enthalpy-porosity approach considers latent heat storage by presenting a latent heat term to the computational grid elements in the energy equation based on its temperature. When the PCM in each grid cell undergoes a phase transition, it is modelled as a porous medium, and the medium porosity is the melt fraction of the PCM within the medium. The constant for the mushy zone was set at 10^5^. The governing equations adopted in this study are shown as the following.

1. Mass Conservation equation.
  12 $$\frac{\partial {V}_{x}}{\partial x}+\frac{\partial {V}_{y}}{\partial y}+\frac{\partial {V}_{z}}{\partial z}=0$$
  where: $velocity vector=({V}_{x},{V}_{y},{V}_{z})$, ${V}_{x}$: velocity in the x-axis direction, ${V}_{y}$: velocity in the y-axis direction, and ${V}_{z}$: velocity in the z-axis direction.
2. Momentum conservation equation.
  - x-direction
    13 $$\rho \left(\frac{\partial {V}_{x}}{\partial t}+{V}_{x}\frac{\partial {V}_{x}}{\partial x}+{V}_{y}\frac{\partial {V}_{x}}{\partial y}+{V}_{z}\frac{\partial {V}_{x}}{\partial z}\right)=\mu \left(\frac{{\partial }^{2}{V}_{x}}{\partial {x}^{2}}+\frac{{\partial }^{2}{V}_{x}}{\partial {y}^{2}}+\frac{{\partial }^{2}{V}_{x}}{\partial {z}^{2}}\right)-\frac{\partial p}{\partial x}+{S}_{x}$$
  - y-direction
    14 $$\rho \left(\frac{\partial {V}_{y}}{\partial t}+{V}_{x}\frac{\partial {V}_{y}}{\partial x}+{V}_{y}\frac{\partial {V}_{y}}{\partial y}+{V}_{z}\frac{\partial {V}_{y}}{\partial z}\right)=\mu \left(\frac{{\partial }^{2}{V}_{y}}{\partial {x}^{2}}+\frac{{\partial }^{2}{V}_{y}}{\partial {y}^{2}}+\frac{{\partial }^{2}{V}_{y}}{\partial {z}^{2}}\right)-\frac{\partial p}{\partial y}+{S}_{y}$$
  - z-direction
    15 $$\rho \left(\frac{\partial {V}_{z}}{\partial t}+{V}_{x}\frac{\partial {V}_{z}}{\partial x}+{V}_{y}\frac{\partial {V}_{z}}{\partial y}+{V}_{z}\frac{\partial {V}_{z}}{\partial z}\right)=\mu \left(\frac{{\partial }^{2}{V}_{z}}{\partial {x}^{2}}+\frac{{\partial }^{2}{V}_{z}}{\partial {y}^{2}}+\frac{{\partial }^{2}{V}_{z}}{\partial {z}^{2}}\right)-\frac{\partial p}{\partial z}+{S}_{z}$$
    where: the source terms ${S}_{x}, {S}_{y}, {S}_{z}$ control the flow in the mushy domain. Under the presence of source terms, the momentum equations obey Carman-Kozeny equations for flow in a porous medium^54^. The gravity force terms in the momentum equations have been omitted as the present study is conducted under zero gravity conditions. The gravity terms in the momentum equation current on the right-hand side with a positive sign as $(\rho {g}_{x}),(\rho {g}_{y}),\mathrm{and} \left(\rho {g}_{z}\right)$ in x-direction, y-direction, and z-direction, respectively.
    16 $${S}_{x}= -{A}_{mushy}\frac{{(1-\mathrm{F})}^{2}}{{\mathrm{F}}^{3}+b}{V}_{x}$$
    17 $${S}_{y}= -{A}_{mushy}\frac{{(1-\mathrm{F})}^{2}}{{\mathrm{F}}^{3}+b}{V}_{y}$$
    18 $${S}_{z}= -{A}_{mushy}\frac{{(1-\mathrm{F})}^{2}}{{\mathrm{F}}^{3}+b}{V}_{z}$$
    where: ${A}_{mushy}$ is a constant related to the mushy zone and varies from 10^4^ to 10^6^, $F$ is the PCM melt fraction, and $b$ is a small number greater than zero for avoiding division over zero, and floating-point errors were adopted at 0.002. The melt fraction was temperature dependent, as shown in Eq. (19).
    19 $$\mathrm{F}=\left\{\begin{array}{ll}0 & T<{T}_{solid}\\ \frac{{T-T}_{solid}}{{T}_{liquid}-{T}_{solid}} & {T}_{solid}<T<{T}_{liquid}\\ 1 & T>{T}_{liquid}\end{array}\right.$$
    where: ${T}_{liquid}$*:* is the liquid temperature, ${T}_{solid}$: is the solid temperature. The difference between them was supposed to be 1 ℃.

3. Energy conservation equation
  20 $$\rho \left(\frac{\partial H}{\partial t}+\frac{\partial (uH)}{\partial x}+\frac{\partial (vH)}{\partial y}+\frac{\partial (wH)}{\partial z}\right)=\frac{\partial }{\partial x}\left(k\frac{\partial T}{\partial x}\right)+\frac{\partial }{\partial y}\left(k\frac{\partial T}{\partial y}\right)+\frac{\partial }{\partial z}\left(k\frac{\partial T}{\partial z}\right)+{S}_{h}$$

The total enthalpy H is defined as shown in Eq. (21).

21 $$H=h+\nabla H$$

22 $$h={h}_{ref.}+{\int }_{{T}_{0}}^{T}{C}_{p} dT$$

23 $$\nabla H=\mathrm{F}L$$

where L: is the latent heat and $\nabla H$: is melting heat. ${S}_{h}$: is the enthalpy source. ${h}_{ref.}$: is a reference enthalpy at reference temperature such that:

24 $${S}_{h}=\frac{\partial \rho \left(\nabla H\right)}{\partial t}+div\left(\rho U\nabla H\right)$$

The latent enthalpy is a piecewise polynomial in which the temperature is the independent variable.

25 $$\nabla H=\left\{\begin{array}{l}L T>{T}_{iquid}\\ \frac{{T-T}_{solid}}{{T}_{liquid}-{T}_{solid}}L{T}_{solid}<T<{T}_{liquid}\\ 0 T<{T}_{solid}\end{array}\right.$$

Pressure–velocity coupling was implemented by utilising a simple scheme in the numerical model. Pressure and energy were spatially discretised using the pressure staggering options (PRESTO) and second-order upwind approaches. The continuity, momentum, and energy equation convergence criteria were 10^–5^, 10^–5^, and 10^–7^, respectively. The relaxation factors for pressure, momentum, liquid fraction, and energy were 0.3, 0.7, 0.9, and one, respectively.

## Results and discussions

The results of the present work are explained in this section. The findings of the grid and time-step independence investigations are presented in “Mesh and time-step independence tests”. Additionally, the current numerical model was validated using earlier published experimental research, and the validation findings are discussed in “Numerical model verification”. In addition, “The effects of the number of fins” outlines how the number of fins affects thermal performance. The implications of pin fin geometry are shown in “The effects of fins geometry”.

### Mesh and time-step independence tests

For the current model, the influence of the grid element density and the time step value of the transient solution was precisely investigated. ANSYS meshing was used to produce the mesh. The employed mesh control methods were: multizone and body sizing. The former was used to construct cell zones associated with a high percentage of hexagonal elements. At the same time, the latter was employed to regulate the size of the computational domain elements. Grid and time-step independence were investigated using an RT 35 PCM operating at 20 W. Four cases of mesh quality at various element densities were studied and reported in Table 2**.**

**Table 2:** Mesh and time-step independence test results.

Mesh quality	Mesh independence test			Time step independence test	
	Number of grid elements	Maximum temperature (K)	Melt fraction at the end of the heating process	Time step (s)	Peak temperature (K)
Coarse	21,617	315.74	0.711	5	315.74
Medium	145,650	315.28	0.720	2	315.35
Fine	765,232	315.13	0.722	1	315.25
Extremely fine	1,074,880	315.04	0.723	0.1	315.19

**Figure 7:**
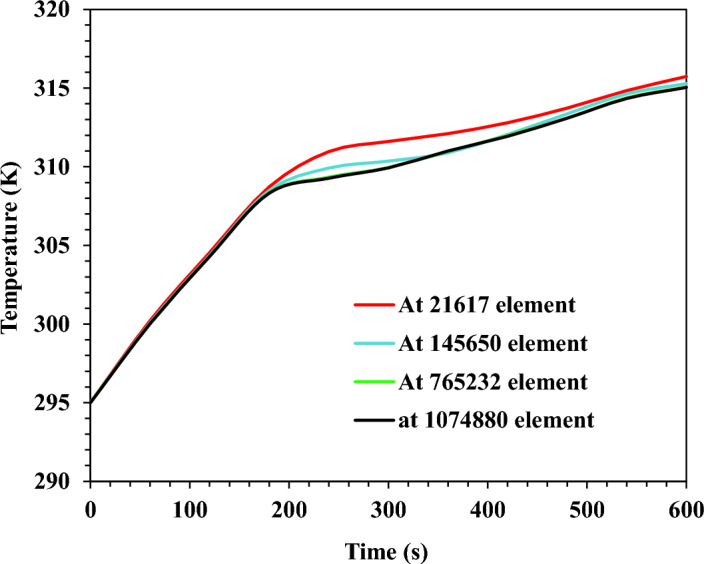
Results of grid independence study for TCD with square fins at $\Psi$ = 20% and N = 80 fins.

The grid independence test findings indicated that improving the mesh quality led to a good convergence of the results. The most considerable inaccuracy observed for estimating the maximum base plate temperature was 0.19% and 0.028% between the coarse and fine meshes at the fine and extremely fine meshes, respectively. The extremely fine mesh was adopted in the current work for the accuracy of the results and the ability to capture the unclear distinctions between the case studies. Figure 7 shows the transient base plate temperature results at different mesh counts. It clearly shows a remarkable convergence between the extremely fine mesh of 1,074,880 elements and the fine mesh of 765,232 elements.

A time-step independence test was performed based on the extremely fine mesh quality. The proper time step must be chosen for allocating the phase change process, as the greater time steps may allow for skipping the entire phase transition process. The time steps shown in Table 2 were the solver's maximum values. The time step values adopted by the solver may be lower than the maximum values to meet the convergence criteria. The time step adopted varied from 5 to 0.1 s, and the independence test showed no discernible change in the maximum temperature of the base plate. The highest error obtained between the time step of 5 s and 0.1 s was 0.17%. Therefore, a time step of 5 s was chosen to compromise between the solution speed and finding accuracy. The adopted time steps were also examined for each mesh size adopted in the present work (coarse, medium, fine, and extremely fine mesh). No significant change in the temperature error was detected, as mentioned in Table 2**,** at the extremely fine mesh.

### Numerical model verification

The current model was verified using Yang et al.^50^ experimental study. The experimental investigation examined fin arrangement's influence on a heat sink's thermal performance. Three different fin configurations were investigated: 1 × 1 cross fins, 2 × 2 cross fins, and without fins. Two PCMs, E-BiInSn and Octadecanol, were examined with a constant volume of 100 ml. The heat sink was made of 6063 aluminium alloy, and its dimensions were 80 × 80 × 30 mm from the outside and 72 × 72 × 25 mm from the inside. Fin thickness was 2 mm in all models. Four heating rods were inserted in a copper block with an exterior size of 60 × 60 × 20 mm and were affixed to the device base plate as the heating source. Three heating loads of 320 W, 200 W, and 80 W were applied to the device base plate. The heat sink was subjected to the heat load until its base plate reached 140 °C. The numerical model verification test was performed utilising E-BiInSn and Octadecanol in the heat sink configuration with two cross fins and nine cavities. The heat load was delivered to the copper block's bottom surface. At the same time, the copper block's sides were insulated.

**Figure 8:**
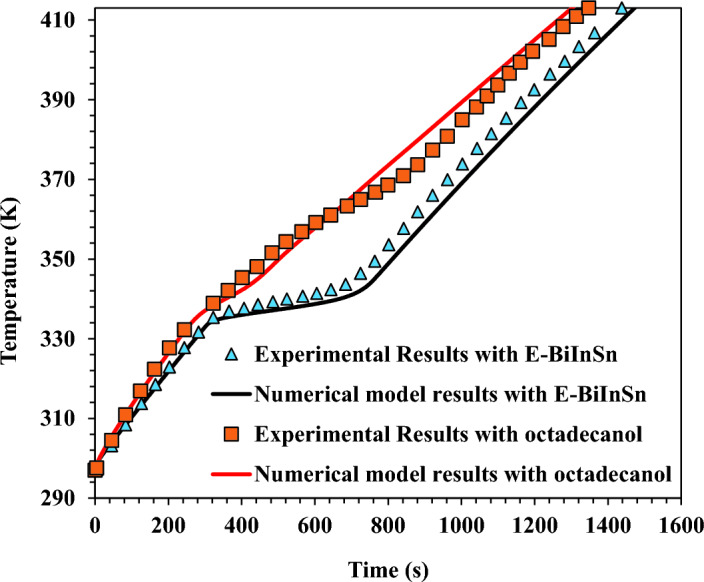
Validation results for the model with the experimental work carried out by Yang et al.^47^ at 80 W heating load.

Additionally, the heat sink's sidewalls and top plate were subjected to heat transfer by convection from the surrounding air. The heat transfer coefficient for convection was 10 W/m^2^ K. The ambient and initial temperature was 297 K. The validation findings in Fig. 8 demonstrate a high degree of matching between the present model and the experimental results obtained by Yang et al.^50^. As seen in Fig. 8, the base plate temperature was kept near constant for an extended period at the melting temperature of each material.

Consequently, the current numerical model successfully captured the phase change phenomenon. The most significant error between the present model and the experimental findings was 1.88%. The latest numerical model was developed using the actual validation setup and solution parameters as the previous one.

The present model was also verified using the experimental work published by Mahmoud et al.^55^. The experimental work tested different configurations of finned heat sinks. Cross-finned and parallel- finned heat sinks were investigated at heating loads of 3 W, 4 W, and 5 W. Parallel fins heat sinks were available in three configurations: three-cavity and six-cavity. In addition, the cross fins arrangement had 9-cavity and 36-cavity configurations. The model was validated against the experimental findings for the 6-cavity heat sink with parallel fins under 3 W and 5 W heating loads. The PCM was made of Rubitherm RT-42 with a melting temperature of 42 °C. In contrast, the heat sink structure was made of Aluminum T6-6061.

The heat sink had an exterior width, length, and height of 50 mm, 50 mm, and 25 mm, respectively. By contrast, each hollow was 6 mm wide, 46 mm long, and 23 mm tall. Each fin was 2 mm thick. The PCM volume used was consistent for all the heat sink designs and equalled 25 ml, resulting in a PCM height of 15.09 mm in the heat sink with parallel fins and six cavities. For 60 min, 4 W and 3 W heat loads were supplied at the top plate. The heat sink side walls were also insulated adequately.

**Figure 9:**
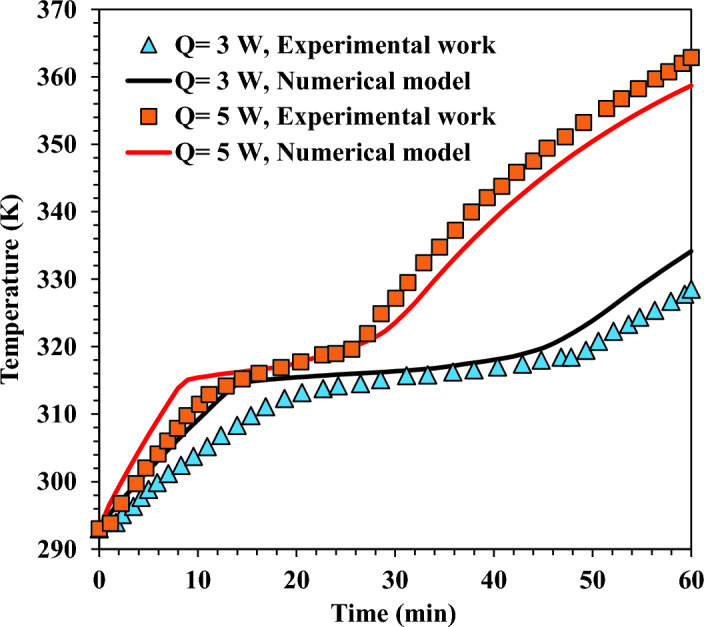
Validation results for the numerical model with the experimental work conducted by Mahmoud et al.^52^.

Meanwhile, the top plate was exposed to convection, and the convective coefficient was 10 W/m^2^ K as adopted in the model. The ambient temperature was adopted as 293 K. In Fig. 9, the transient response was compared between the model and the experimental study of Mahmoud et al.^55^. The findings demonstrate a high matching between the adopted model and experimental results. The numerical model could correctly capture the PCM's phase change phenomenon. Additionally, the numerical model could precisely predict the temperature value of the base plate over time. At 3 W and 5 W, the most significant error between the experimental findings and the numerical model was 1.9% and 1.95%, respectively.

### The effects of the number of fins

This subitem demonstrates the effects of fins number on the base plate temperature, PCM melt fraction, and critical time of heat sink.

#### Temperature results

**Figure 10:**
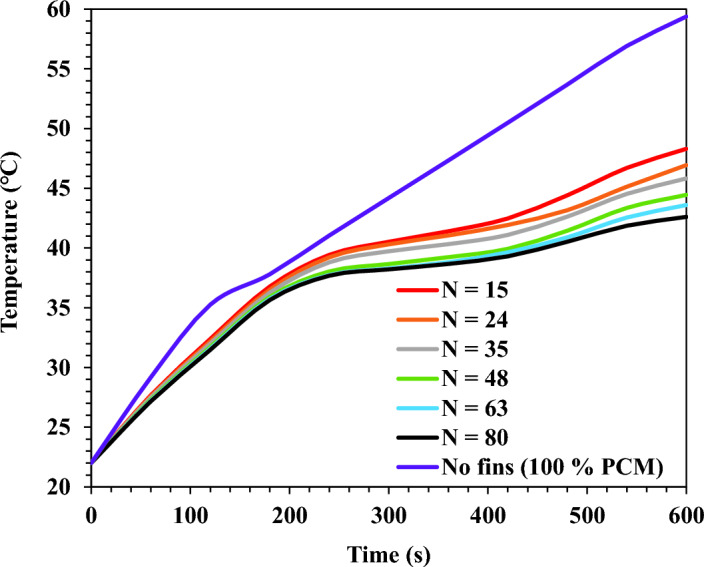
TCD's base plate temperature for square fins at a different number of fins and $\Psi$ = 20% during heating (0 s < t < 600 s).

**Figure 11:**
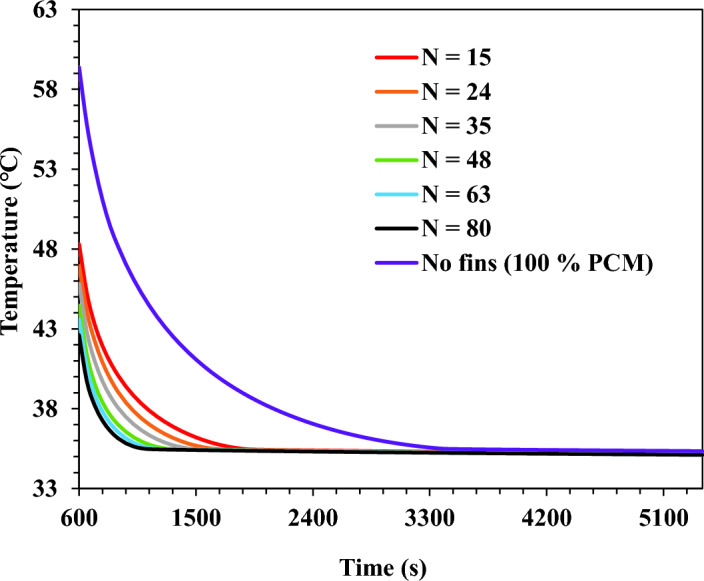
TCD's base plate temperature for square fins at a different number of fins and $\Psi$ = 20% during cooling (600 s < t < 5400 s).

The average base plate temperature was reported for the TCD without and with fins. The TCD with square fins was investigated under varying fins, from 15 fins up to 80 fins, at the same volume fraction of fins. Figs. 10 and 11 show the temperature results at a varying number of fins and $\Psi$ = 20% for the heating and cooling process, respectively.

As shown in Fig. 10, during the heating process (0 s < t < 600 s), the temperature increased from the initial value until it reached its maximum temperature during the heating period. However, Fig. 11 shows the temperature decay during the cooling process. For the cooling process, the temperature decreased until the phase change temperature of the PCM. Therefore, a constant temperature was observed until the end of the cooling process.

As concluded from the findings, the TCD without fins and 100% PCM achieved a maximum temperature of 59.4 ℃. In addition, the temperature values for TCDs with fins were considerably lower than those without fins. The results show a considerable reduction in temperature by increasing the fins number. The TCDs with 80 fins and 15 fins recorded a maximum temperature of 42.6 ℃ and 48.3 ℃ with a reduction percentage of 39.4% and 18.6%, respectively, compared with the case without fins.

#### Melt fraction and critical time results

Thermal analysis was used to record the PCM melt fraction. The volume of liquid PCM as a percentage of the overall volume is called the melt fraction. Results for melt fraction are substantial. As they clearly outline the temperature data for each situation. Higher PCM melt fraction leads to lower temperature outcomes and more remarkable thermal performance improvement for the TCD.

**Figure 12:**
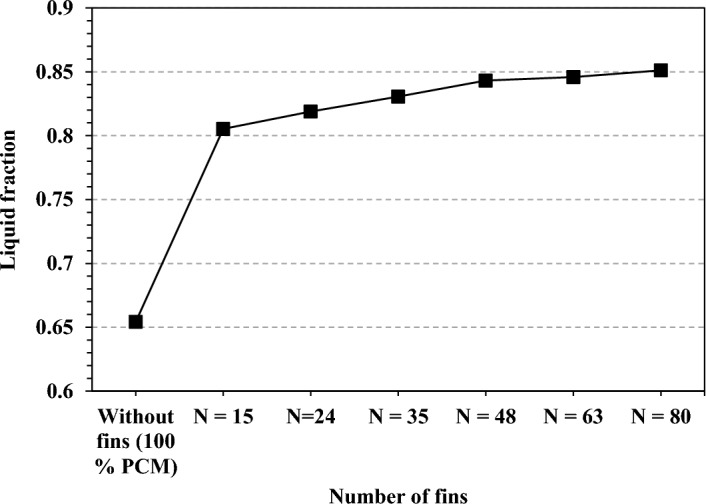
Maximum PCM melt fraction during the cycle at different fin numbers.

Figure 12 shows the maximum melt fraction produced in the cycle for the cases against various fin counts. The maximum PCM melt fraction for the TCD without fins was 0.65. The finned TCDs dramatically raised the melt fraction in contrast. The number of fins was closely related to the melt fraction. The TCD observed a maximum melt fraction of 0.8 with 15-pin fins, an increase of 23% over the case without fins. The maximum melt fraction grew when the number of fins was increased. The results for the melt fraction and temperature were remarkably similar. The maximum melt fraction for the PCM in the TCD with 80-pin fins was 0.85, up 30.8% from the example without fins. As a result, the TCD experienced lower temperatures since more heat was stored as latent heat.

**Figure 13:**
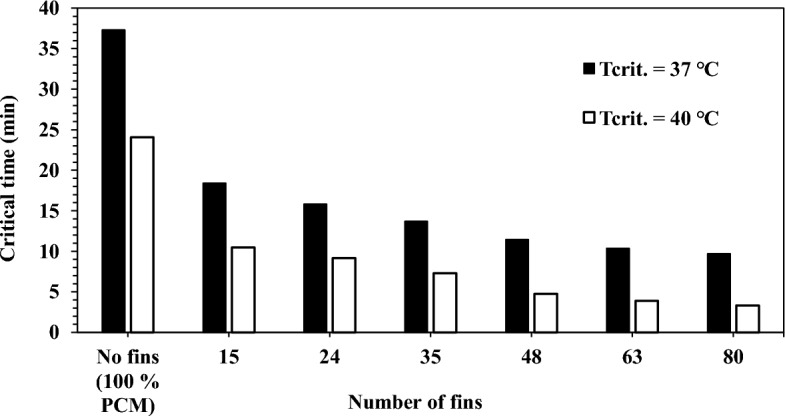
Critical time results at critical temperatures of 37 ℃ and 40 ℃.

The critical time was considered as another parameter for the thermal performance evaluation. Critical time was defined as the time spent by the TCD with temperature values higher than the critical temperatures. Two critical temperatures were considered in the present model results analysis: 37 ℃ and 40 ℃. The critical temperature refers to the maximum temperature the hardware can survive without failure or losing functionality. The critical time results for all cases were plotted against the number of fins in Fig. 13**.**

The findings reported that fins as TCBs reduced the critical time. Further, it was concluded from Fig. 13 that the increase in the fins count decreased the critical time significantly. The TCD without fins recorded a critical time of 37.3 min and 24 min at critical temperatures of 37 ℃ and 40 ℃, respectively. The finned TCD recorded lower values than the TCD without fins. For the TCD of 15 fins, the critical time was 18.4 min and 10.5 min, with a reduction of 50.7% and 56.25% at critical temperatures of 37 ℃ and 40 ℃, respectively. However, the TCD with 80 fins recorded critical times of 9.7 min and 3.3 min, with a reduction percentage of 74% and 86.3% at 37 ℃ and 40 ℃, respectively. The enhanced effect of the TCD with a higher number of fins comes from the fact that dividing the big fins into small fins results in a higher contact area between the PCM and the metallic fins. The lower number of fins has the same mass as the higher number but has a lower contact area with the PCM. In contrast, the higher number of fins have more contact area with the PCM, hence a higher heat transfer rate, lower temperature, and enhanced thermal performance.

### The effects of fins geometry

The effect of fin geometry was examined at two-volume fractions of TCB, 20% and 50%. Six fin configurations were investigated: circular, square, cross-shaped, Triangular, I-shaped and V-shaped. The metrics adopted to measure the thermal performance were the TCD's base plate temperature, the melt fraction of the PCM, and the critical time.

#### The base plate temperature

**Figure 14:**
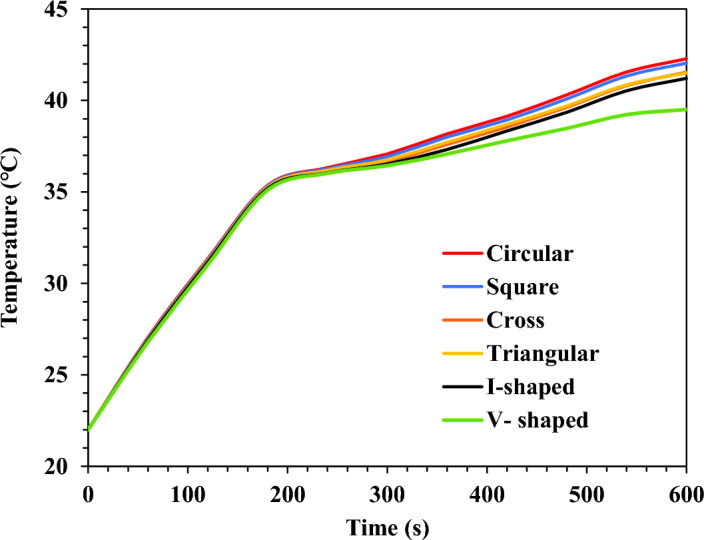
TCD's base plate temperature results at different fin geometries during the heating process (0 s < t < 600 s) at $\Psi$ = 20% and N = 80 fins.

Over its surface, the base plate's temperature was averaged. The transient thermal analysis findings for the six-pin fin designs during the heating process are shown in Fig. 14**.** The TCD's base plate reached its maximum temperature after the heating process. Figure 14 demonstrates how the fin arrangement significantly affected the temperatures and thermal performance. Among the several fin configurations, the geometry of the circular fins recorded the highest temperatures. Fin geometries with square, cross-shaped, and triangular shapes showed improved thermal performance in that order. The base plate temperature was significantly reduced, and the thermal performance was greatly enhanced by the innovative fin geometry of I-shaped and V-shaped.

**Figure 15:**
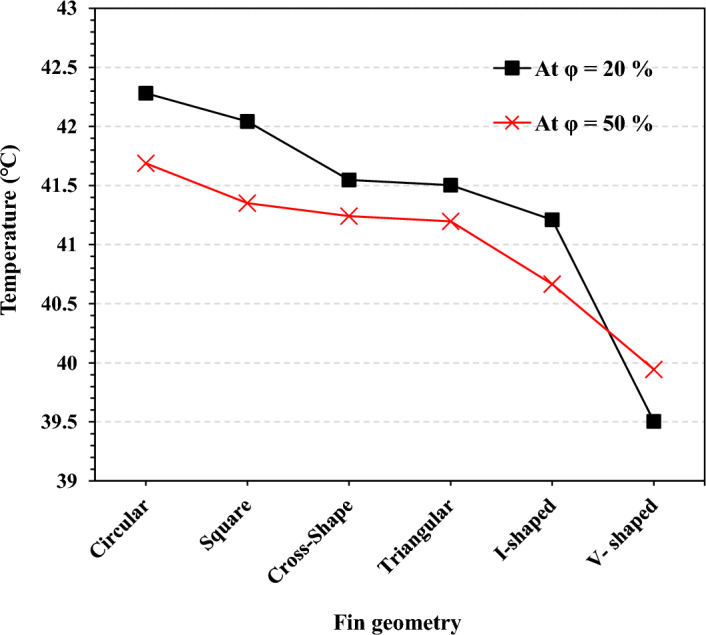
TCD's maximum base plate temperature obtained during the working cycle at $\Psi$ = 20% and $\Psi$ = 50% with N = 80 fins.

Figure 15 shows the maximum base plate temperature obtained during the cycle for the adopted fin configurations at $\Psi$ = 20% and $\Psi$ = 50%. At $\Psi$ = 20%, the circular fins recorded a maximum temperature of 42.3 ℃. At the same time, the square and cross-shape fin geometry recorded lower temperatures of 42 ℃ and 41.54 ℃ with reduction percentages of 0.7% and 1.9%, respectively. The triangular shape obtained a temperature value very close to the value recorded by the cross-shape geometry. The maximum temperatures for I-shaped and V-shaped were 41.2 ℃ and 39.5 ℃, with a reduction percentage of 2.6% and 6.6%, respectively.

At $\Psi$ = 50%, the temperature results for all fin configurations were lower than at $\Psi$ = 20%, except for the V-shaped geometry. Circular fin geometry recorded the highest maximum temperature of 41.7 ℃. At the same time, V-shaped fin geometry recorded the lowest maximum temperature of 39.9 ℃ with a reduction percentage of 4.3%. Unlike the other fin geometries, the V-shaped geometry recorded a base plate temperature at $\Psi$ = 50% higher than the temperature at $\Psi$ = 20%. At $\Psi$ = 50%, all the fin geometries had higher contact surface area, improving the heat transfer coefficient with the PCM. That's why the temperatures were lower at $\Psi$ = 50%. For V-shaped geometry at $\Psi$ = 50%, there was a high degree of interference between the fins, as shown in Fig. 4. This interference between the fins heated the PCM inside this interference quickly, causing the PCM to be melted completely. Thus, any heat gain raised the temperature of the TCD and the base plate.

**Figure 16:**
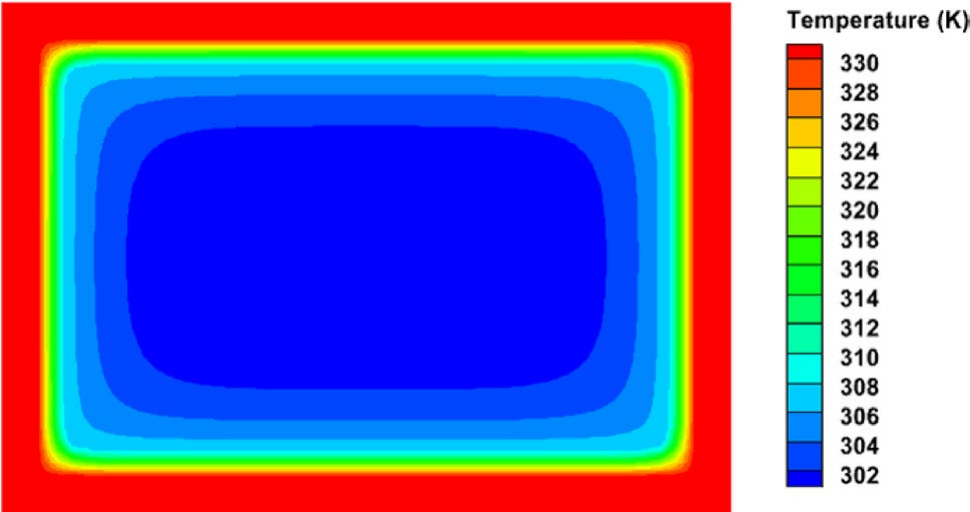
Top view of temperature contour for the TCD without fins (100% PCM).

**Figure 17:**
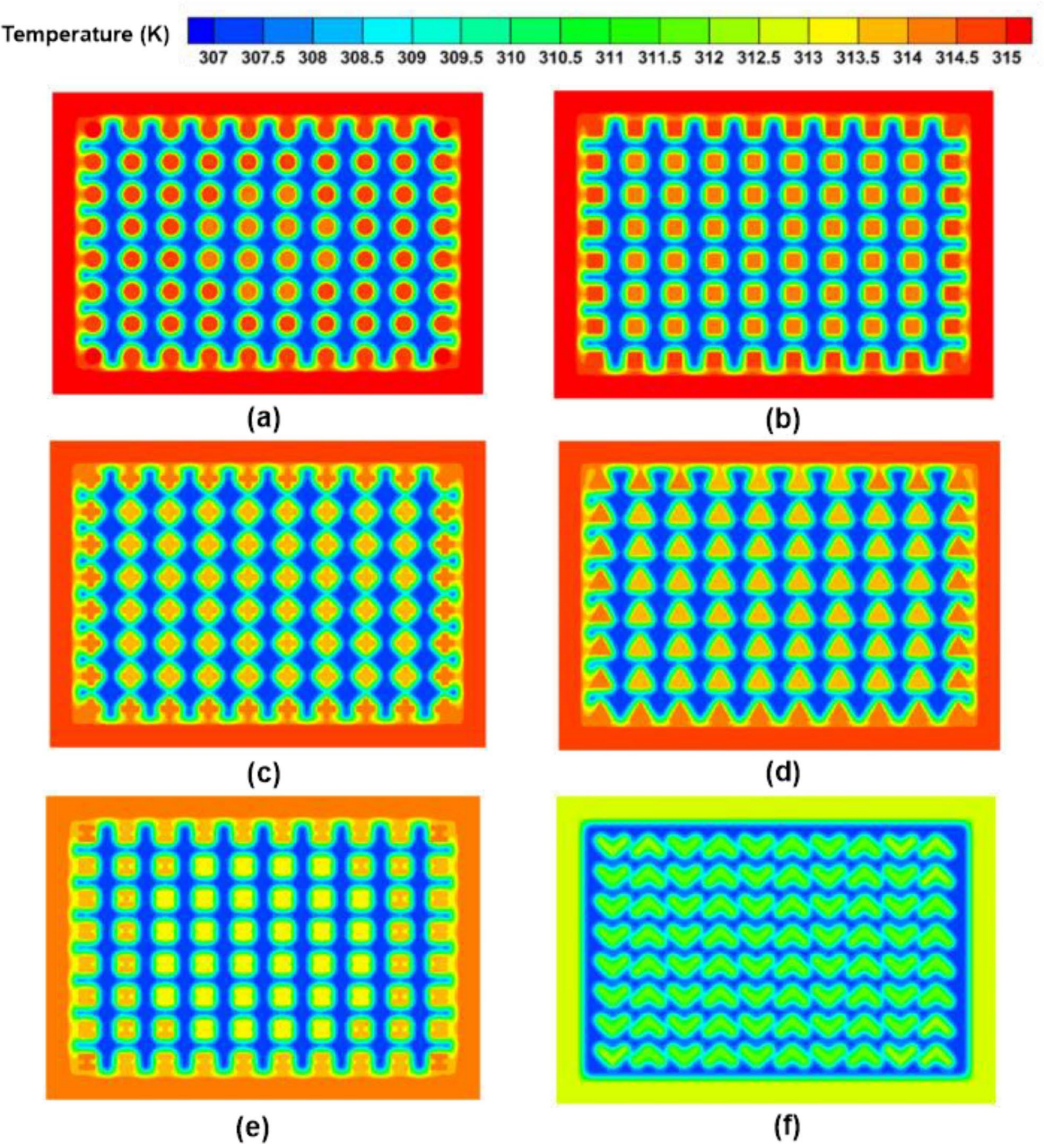
Temperature contours at different fin geometries and $\Psi$ = 20% and N = 80 fins. (**a**) Circular fins, (**b**) square fins, (**c**) cross-shaped fins, (**d**) triangular fins, (**e**) I-shaped fins, and (**f**) V-shaped fins.

Figures 16 and 17 demonstrate the TCD's temperature contours without fins and with fins, respectively. Figure 16 clearly shows that the temperature contour of the TCD without fins was highly affected by the low PCM thermal conductivity. This created a high-temperature gradient inside the PCM in the TCD. The PCM near the surface of the device was heated up to a higher temperature, while the PCM at the centre of the TCD was at a lower temperature. The heated PCM near the TCD walls and base plate quickly melted. Thus, the PCM temperature increased with the heat added, causing a significant increase in the TCD walls' temperature. As a result, the maximum temperature of the heat sink reached 57 ℃ beside the wall, and the minimum temperature was 29 ℃ in the mid of the PCM.

The finned TCDs' temperature contours are shown in Fig. 17. The temperature of the PCM was roughly homogeneous. Additionally, a notable improvement in the PCM's liquid percentage was noted. The circular fins reported the highest temperature and the poorest thermal performance. The V-shaped fin design, in contrast, recorded the highest thermal performance and the coolest TCD temperatures. The V-shaped fins' geometrical and orienting characteristics increased their surface area in contact with the PCM, improving their thermal performance. The integrated fins' enhanced PCM liquid percentage and considerably boosted the heat that could be stored as latent heat. Lower temperature values were, as a result, attained.

**Figure 18:**
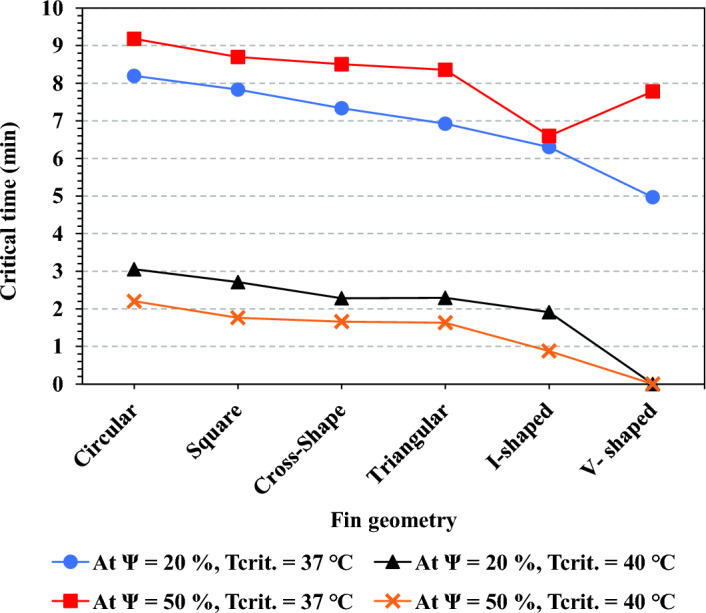
Critical time results for different fins configurations at $\Psi$ = 20% and $\Psi$ = 50% for critical temperatures of 37 ℃ and 40 ℃ at N = 80 fins.

Figure 18 presents the critical time achieved by each fin geometry at critical temperatures of 37 ℃ and 40 ℃ with TCB volume fractions of 20% and 50%. On the one hand, at a critical temperature of 37 ℃, the circular fins achieved the longest critical time of 8.2 min and 9.18 min at volume fractions of 20% and 50%, respectively. On the other hand, at a critical temperature of 40 ℃, the critical time achieved by the circular fins was the longest among the other fin geometries. It achieved 3.1 and 2.2 min at $\Psi$ = 20% and $\Psi$ = 50%, respectively. The V-shaped fin geometry achieved the shorter critical time among all fin geometries. At a critical temperature of 37 ℃, V-shaped fin geometry reduced the critical time by 39.5% and 15.25% at TCB volume fractions of 20% and 50%, respectively. Further, V-shaped fins could prevent the Heat sink from entering the critical zone at a critical temperature of 40 ℃, as shown in Fig. 18 ℃.

**Figure 19:**
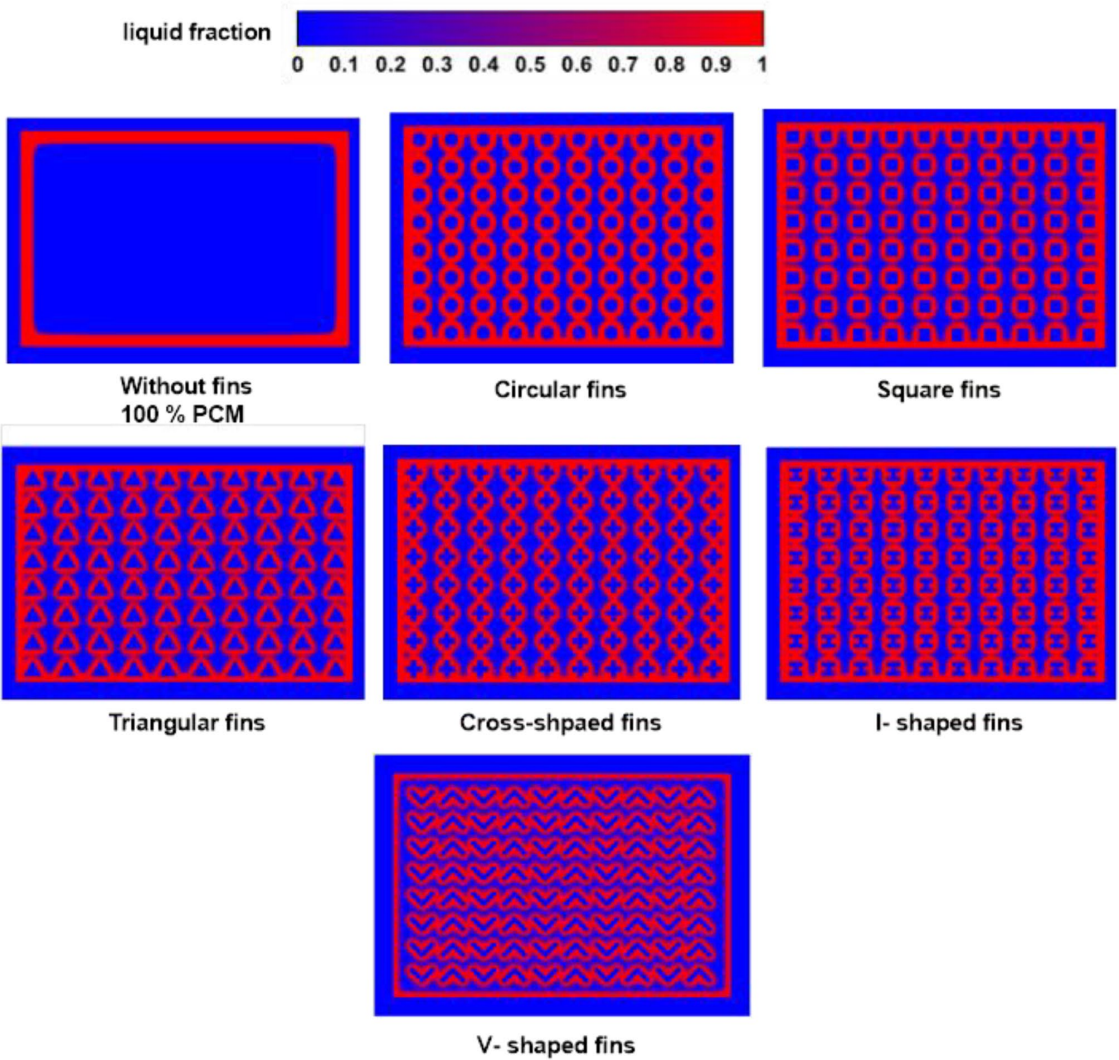
PCM melt fraction contours for the fin configurations at the end of the heating process (t = 600 s) and Ψ = 20% and N = 80 fins.

The PCM's liquid percentage contours at the heating operation's completion are shown in Fig. 19, along with a 20% volume fraction of TCB. The melt fraction measurements were significant since they showed how much latent heat was stored and how well PCM works. The efficacy of the PCM and the thermal performance increase with the amount of PCM that can be melted. Figure 19 makes it abundantly evident that the TCD without fins obtained a lower melt fraction result. The finned TCDs, however, managed to get a significantly greater value. The PCM around the integrated fins, which was heated by conduction through the PCM in the absence of fins, significantly raised the melt fraction values. The circular fins of the finned TCD produced the lowest melt fraction. However, the geometry of the V-shaped fins showed the highest melt fraction.

**Figure 20:**
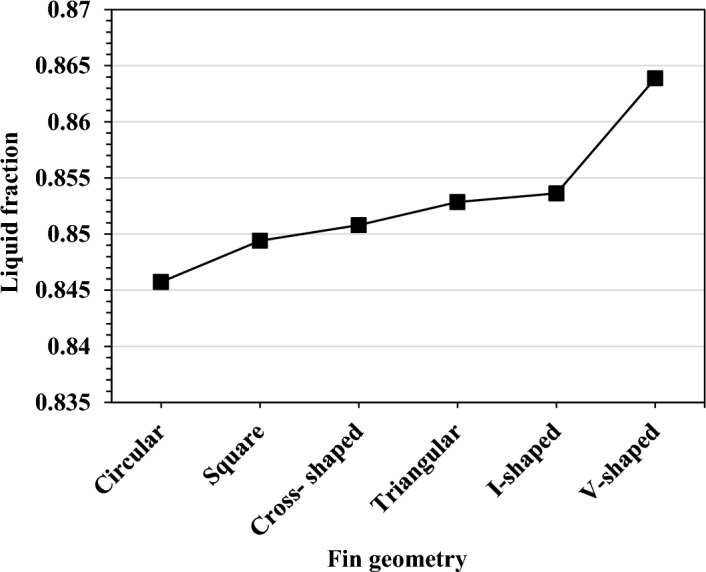
Maximum PCM melt fraction obtained with fins configurations at Ψ = 20% and N = 80 fins.

The highest melt fraction for the PCM during the cycle at a 20% TCB volume fraction is shown in Fig. 20. The PCM melt fraction in the TCD with 100% PCM and no fins was 0.65. The circular fins reported a maximum melt fraction of 0.85 with an improvement percentage of 30.7% for the cases with a finned TCD. The best melt fraction result was 0.86, with an increasing percentage of 32.3% for the V-shaped fin design. The TCD's base plate temperature values for the V-shaped fin design agreed with the melt fraction findings. The PCM melts more due to the excellent contact between the V-shaped fins and the PCM. As a result, more of the provided heat was stored as latent heat without raising the heat sink temperature. At 50% TCB volume fraction, The circular fin configuration had the lowest melt fraction of 0.98 among the other configurations. However, the maximum melt fraction of unity was measured in the V-shaped design. The difference in the contact area between the TCD walls and the PCM in the following subsection explains why each fin geometry's thermal performance differs.

### The enhancement in the contact area

**Figure 21:**
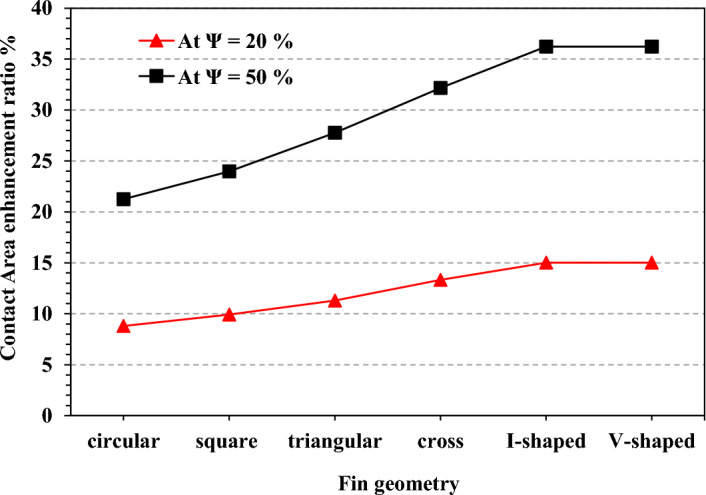
Contact area enhancement ratio for all pin fin configurations at N = 80 fins.

Figure 21 shows the results of the contact area enhancement ratio between PCM and fins at TCB volume fractions of 20% and 50%. The contact area refers to the surface area of the TCD structure, which is in direct contact with the PCM. The greater the contact area, the greater the heat transfer enhancement from the TCD structure to the PCM. The pin fins integrated into the TCD structure significantly increased the contact area and enhanced the TCD's thermal performance. It was provided that the novel pin fin geometries of cross-shaped, I-shaped, and V-shaped significantly improved the contact area with the PCM. The contact area explains the better thermal performance of such novel pin fin geometries. At a TCB volume fraction of 50%, the circular pin fins enhanced the contact area by about 12.45%.

Contact area of the novel pin fins was significantly higher. At the same time, Cross-shaped fin geometry recorded an enhancement ratio of 18.8%. At the same time, the I-shaped and V-shaped recorded the same enhancement ratio of 21.1%. At 20% TCB volume fraction, the contact area was reasonably lower than the TCB volume fraction of 50% as the area of the fins' surface was lower. The circular fin geometry recorded an enhancement ratio of 8.8%. The I-shaped and V-shaped fins recorded the highest ratio of 15%. It seems a bit confusing that although the I-shaped and V-shaped geometry recorded the same contact area enhancement ratios, the V-shaped geometry's thermal control performance was remarkably better than the I-shaped geometry. The explanation is that the V-shaped fins were arranged better than the I-shaped fins in the heat sink cavity. In addition, there was a degree of homogeneity and sound interference for the PCM material around each fin.

Future work may be dedicated to investigating the effect of fin arrangement and orientation inside the TCD on its thermal management performance.

## Conclusion

The thermal environment the small satellites are exposed is highly intermittent, which imposes severe challenges to satellite subsystems' thermal control design. The present study adopted a PCM-integrated TCD to manage satellite subsystems in space. Metallic pin fins are a common method for enhancing the PCM's low thermal conductivity and were adopted in our study. The effects of pin fin geometry and the fin number were carefully examined. The pin fins were designed with two volume fractions: 20% and 50%. The TCD model consisted of a heat sink made from aluminium due to its relatively high thermal conductivity. During a phase change, PCM was inserted inside the TCD to take advantage of latent heat storage. Six-pin fin geometries were integrated into the TCD cavity. The conventional pin fins' geometries of circular, square, and triangular. Then the novel geometries of cross-shaped, I-shaped, and V-shaped. The numerical model was carefully validated with previous experimental studies to ensure the reliability of the results.

The results of the present research concluded that:

1. Increasing the number of integrated fins while maintaining a constant volume fraction of fins enhanced the thermal performance significantly. The TCD's base plate maximum temperature was reduced by 18.68% at 15-pin fins. However, the drop was 39.44% at 80 fins compared to the situation without fins.
2. Pin fin geometry and their arrangement inside the TCD significantly affect the thermal performance. The pin fins greatly enhanced the thermal performance by substantially increasing the PCM's melt fraction.
3. The higher the pin fin volume fraction, the higher the contact area between the PCM and fins, hence better thermal performance. The fin volume fraction Ψ = 50% performed better than Ψ = 20%.
4. The novel pin fin geometries of I-shaped and V-shaped enhanced the thermal performance of the TCD significantly.
5. Novel V-shaped pin fin geometry recorded the best thermal performance and could decrease the TCD's base plate maximum temperature by about 6.6% and 4.3% at TCB volume fractions of 20% and 50%, respectively. In addition, the V-shaped geometry could reduce the critical time remarkably among the fin geometry cases.
6. The interface area between the fins and the PCM enhanced the PCM thermal conductivity significantly. The novel pin fin geometry of I-shaped and V-shaped could improve the contact area significantly.

## Supplementary Information

Supplementary Information.

## Data Availability

The datasets used and/or analysed during the current study are available from the corresponding author upon reasonable request.

## English symbols

*A*_*s*_: The surface area (m^2^)
*C*_*P*_: Specific heat (J/kg. K)
*A*_*Fins*_: Fins side area (m^2^)
*A*_*Walls*_: Walls surface area of TCD (m^2^)
*H*: Enthalpy (J/kg)
*H*_*s*_: Sensible enthalpy (J/kg)
*H*_*l*_: Latent enthalpy (J/kg)
*HC*: Hydrocarbons
*k*: Thermal conductivity (W/m. K)
*L*: Latent heat (J/kg)
*N*: Number of fins
*q*: Heat flux (W/m^2^)
*Q*: Heat load (W)
*t*: Time (S)
*T*: Temperature (K)

## Greek symbols

*ρ*: The density (kg/m^3^)
*α*: Heat absorptivity
*ε*: Heat emissivity
*σ*: Stefan–Boltzmann constant (W/m^2^ K)
*μ*: The dynamic viscosity (kg/m s)
*φ*: Fins’ volume fraction
*Ψ*: PCM melt fraction

## Abbreviations

EG: Expanded graphite
LEO: Low earth orbit
LES: Latent energy storage
MWCNTs: Multi-wall carbon nanotubes
PCM: Phase change material
SES: Sensible energy storage
TCB: Thermal conductivity booster
TCD: Thermal control device
TCS: Thermal control subsystem
TCU: Thermal control unit
TES: Thermal energy storage materials
TM: Thermal management
TVC: Thermal vacuum chamber

## Subscripts

*L*: Latent
*M*: Melting
*max*: Maximum
*Rad*: Radiation
*Ref*: Reference
Sen.: Sensible
t: Total
∞: Infinity (surrounding temperature)
i: Initial
