# Supplementary material for: Boosting the thermal management performance of a PCM-based module using novel metallic pin fin geometries: Numerical study
Source: Sci Rep. 2023 Jul 6;13:10955. doi: 10.1038/s41598-023-37639-3 (PMC10326248; doi:10.1038/s41598-023-37639-3)
Supplement: Supplementary file 1 — Supplementary Information. [file 41598_2023_37639_MOESM1_ESM.docx]

**Appendix (A).**

This appendix includes illustrations of the heat sink's precise dimensions and fin arrangement. The heat sink was 100 mm long, 70 mm wide, and 20 mm tall on the outside. The configuration of the traditional fin geometries is shown in Figure A (1), whereas the innovative fin geometries is shown in Figure A (2). Table A (1) illustrates the equation for each fin geometry used for calculating the fin dimensions. Table A (2) lists the fundamental measurements for each fin configuration shown in Figures A (1) and A (2). Equation A (1) presents the equation used for calculating the fin cross section dimensions based on the fin fraction $\Psi$.

$\boldsymbol{\Psi}\boldsymbol{=}\frac{\boldsymbol{n}\boldsymbol{A}_{\boldsymbol{c}}\boldsymbol{L}}{\boldsymbol{V}_{\boldsymbol{PCM}}}$ **Eq. A (1)**

Where n is number of fins, $A_{c}$ is the cross-sectional area of fin and L is the fin length.

**Table A (1): The equations corresponding to each fin geometry used for calculating the fin dimensions.**

| Fin geometry | Circular | Square | Triangular | Cross-shaped | I-shaped | V-shaped |
| --- | --- | --- | --- | --- | --- | --- |
| Equation | $\Psi=\frac{n\pi a^{2}L}{4V_{PCM}}$ | $\Psi=\frac{na^{2}L}{V_{PCM}}$ | $\Psi=\frac{n\sqrt{5}a^{2}L}{{4V}_{PCM}}$ | $\Psi=\frac{n5a^{2}L}{V_{PCM}}$ | $\Psi=\frac{n7a^{2}L}{V_{PCM}}$ | $\Psi=\frac{n7a^{2}L}{V_{PCM}}$ |

**Table A (2): The dimensions of the heat sink for all fin geometries**

**at TCB volume fractions of 20% and 50%.**

| Fin geometry | Circular | | Square | | Triangular | | Cross-shaped | | I-shaped | | V-shaped | |
| --- | --- | --- | --- | --- | --- | --- | --- | --- | --- | --- | --- | --- |
| φ | 20% | 50% | 20% | 50% | 20% | 50% | 20% | 50% | 20% | 50% | 20% | 50% |
| a (mm) | 3.78 | 5.35 | 3.35 | 4.74 | 5.1 | 7.4 | 1.5 | 2.12 | 1.267 | 1.79 | 1.26 | 1.79 |
| S (mm) | 3.72 | 2.15 | 4.15 | 2.76 | 3.1 | 1.26 | 3 | 1.14 | 3.7 | 2.13 | 6.8 | 6.8 |
| L (mm) | 5.22 | 3.65 | 5.65 | 4.26 | 3.91 | 1.79 | 4.5 | 2.64 | 5.12 | 3.63 | 8.18 | 8.18 |


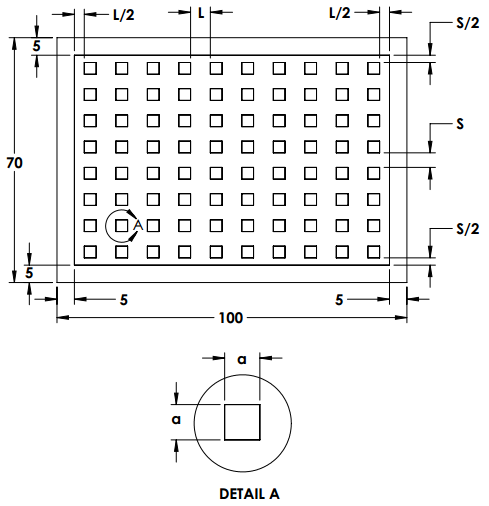

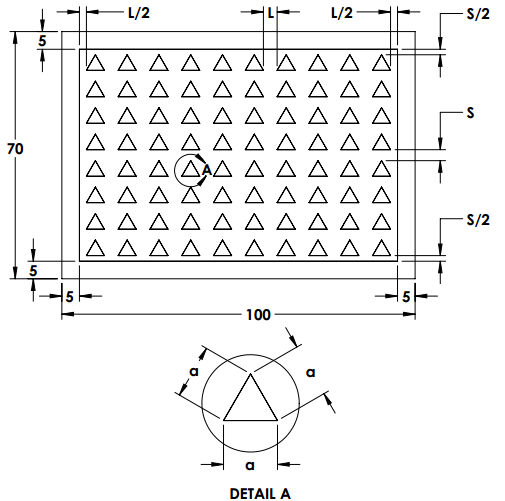

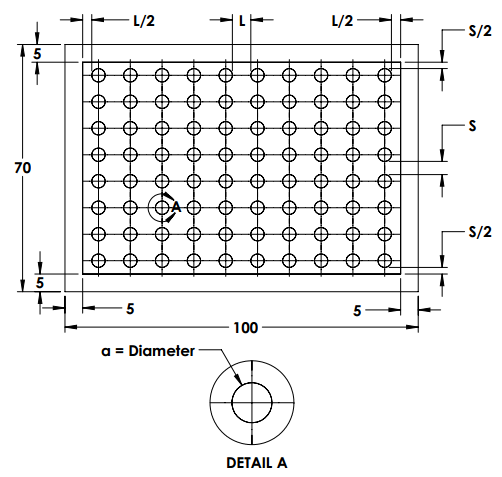


**Fig. A (1): The heat sink configuration with conventional pin fin geometries**


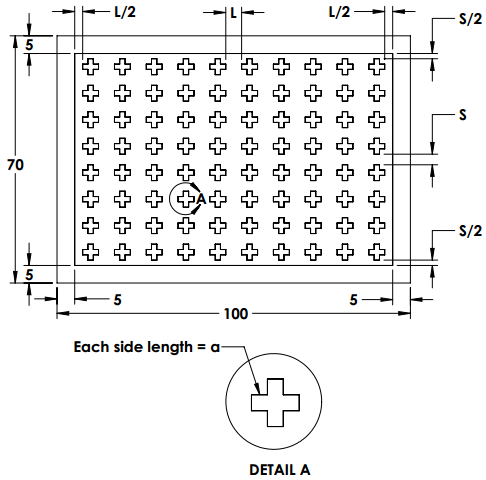

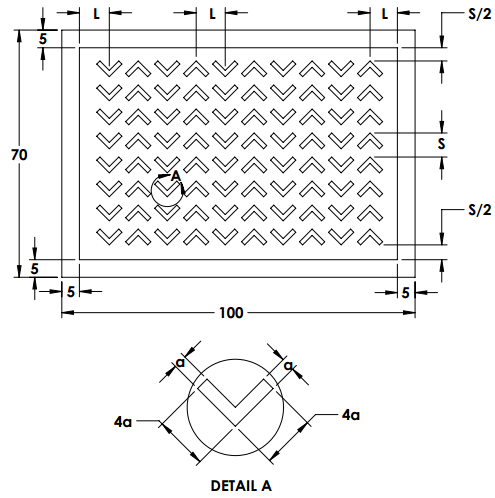

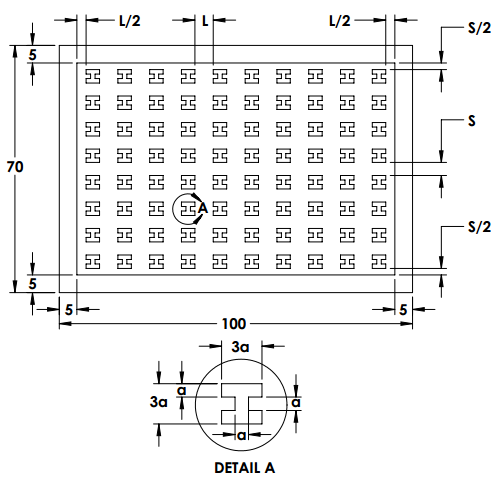


**Fig. A (2): The heat sink configuration with novel pin fin geometries**
